# Micro-remediation of chromium contaminated soils

**DOI:** 10.7717/peerj.6076

**Published:** 2018-12-05

**Authors:** Hadia -e- Fatima, Ambreen Ahmed

**Affiliations:** Department of Botany, University of the Punjab, Lahore, Pakistan

**Keywords:** Auxin, Soil polluion, *Lens culinaris*, Rhizobacteria

## Abstract

Bacteria are tiny organisms which are ubiquitously found in the environment. These microscopic living bodies are responsible for the flow of nutrients in biogeochemical cycles and fertility imparted to the soil. Release of excessive chromium in agricultural soils due to rapid growth of industries may result in minimizing the fertility of soil in future, which will lead to reduction in crop production. Plant growth promoting bacteria (PGPB) are beneficial to the environment, some of which can tolerate chromium and protect plants against heavy metal stress. The current study aims to identify such chromium-tolerant auxin-producing rhizobacteria and to investigate their inoculation effects on the growth characteristics of *Lens culinaris* in chromium polluted soils by using two different chromium salts i.e., K_2_Cr_2_O_7_ and K_2_CrO_4_ in varying concentrations (0, 50, 100, 200, 400 and 500 µgml^−1^). The results revealed that *Bacillus* species are efficient in significantly reducing the deleterious effects of Cr. These effective bacterial strains were able to stimulate the growth of metal effected plants of *Lens culinaris* which were grown in chromium contaminated environment. Therefore, these plant growth promoting rhizobacteria PGPRs, having both auxin production potential and chromium-resistance ability, are considered as efficient micro-factories against chromium pollution.

## Introduction

Microorganisms living in the rhizospheric regions are better able to naturallly degrade hazardous soil compounds into less toxic forms. Beneficial interactions of these microscopic creatures with soil nutrients are essential for a balanced nutrient flow in the soil. Therefore, being primary producers, bacteria play an important role for sustaining life on earth (Ma et al., 2015).

Rapid industrialization over the past many years has resulted in many environmental problems worldwide (Costa & Klein, 2006; Sankhla et al., 2016; Singh et al., 2017). As a result of industrialization, heavy metal pollution has become a serious issue in developing countries including Pakistan. The major problems which are caused by accumulation of these toxic metals in soil and soil sediments include lowering of soil fertility, lowering of crop yield, and quality of agricultural products (Lin et al., 2018; Ontañon et al., 2018; Parewa et al., 2018). It also exerts negative impact on human life quality, animals and plants by penetrating into the food chains (Srivastava et al., 2018). Pollution due to heavy metal contamination deteriorates the quality and production of crops in nearby soils and water bodies, thus threatening the existence of living organisms on earth. The most common human activities which are a potential source of this heavy metal contamination in water, air and soils are industrial effluents, disposal of sewage sludge, atmospheric deposition of pollutants released from industrial processes, domestic and industrial wastes, mining activities, use of agrochemicals and land fill operations. Release of these carcinogenic contaminants from various industrial sources, sewage sludge and agrochemicals poses a major threat to the soil environment. Generally, heavy metals are not degraded naturally and persist in the environment indefinitely. Therefore, remediation of metal-contaminated soils becomes important, as they become incompatible for sustainable agriculture.

Many reports suggest that microbial activities may counteract negative effects of chromium (Cr) and other heavy metals (HMs) in plants growing in contaminated areas through certain mechanisms (Jayaprakashvel et al., 2017). These microorganisms produce phytohormones and stimulate plant growth in these polluted soils. Therefore, these plant-bacterial interactions are quite helpful for macro- and micro-partners i.e., green plants and plant-associated bacteria (Mohapatra et al., 2017). Rhizoremediation is one promising approach which utilizes these beneficial microbes to lower the metal toxicity in chromium polluted soils. This utilization of bacterial species relies upon triangular interactions between microbes, plants and pollutants (it makes a triangle between plant, microbes and pollutants) where chemical exudates released in rhizospheric region by plant roots help to stimulate bacterial activity to enhance chemical and physical properties of soil and to increase metal detoxification, nutrient acquisition and alleviation of abiotic stress in plants. Such metal-mobilizing microscopic entities can become potential candidates for rhizoremediation since these bacterial species produce various metabolites, including organic acids, various biosurfactants and siderophores thus increasing the number of bioavailable essential nutrients in the rhizosphere and make them viable for plant uptake. Similarly, the efficacy of remediation process may also be enhanced by the use of chromium-tolerant plant growth-promoting bacteria (PGPB) as beneficial inoculants–which reduce metal toxicity by increasing plant nutrient acquisition and also improve plant vigor and biomass production under adverse environmental conditions (Francisco et al., 2018). These chromium-tolerant auxin-producing rhizobacteria may prove to be an efficient group of bacteria that are involved in active colonization by plant roots resulting in increase in plant growth and yield by reducing chromium toxicity in chromium contaminated areas (Feng et al., 2016). In the industrial processes, various Cr-salts are used. The current study proves that K_2_Cr_2_O_7_ is more toxic than K_2_CrO_4_ so, wherever possible in the industries, it should be replaced with other Cr-salts like K_2_CrO_4_ which are less toxic.

## Experimental Procedures

### Screening of chromium-tolerant auxin-producing isolates and their characterization

In the present study, different soil and water samples were collected from contaminated areas of Kasur, Pakistan. MIC (minimum inhibitory concentration) of chromium salt stress for the growth of selected bacterial isolates was determined using K_2_Cr_2_O_7_ and K_2_CrO_4_. Bacteria which were resistant to chromium, were then screened for their auxin production potential. Three chromium-tolerant auxin-producing bacterial strains (EII, 3a, EIV) were selected and characterized for their morphological as well as physiological characteristics. Molecular identification of these bacterial isolates was carried out using 16S rDNA sequencing and obtained DNA sequences were then searched for their homology with other sequences through BLAST and submitted to GenBank to obtain their accession numbers.

### Interactions of bacterial isolates with *Lens culinaris*

Three chromium-tolerant auxin-producing isolates (EII, 3a and EIV) were used to treat *Lens culinaris* (var. masoor-2009) seeds with and without chromium stress. The effect of selected bacterial cultures with and without chromium stress (K_2_Cr_2_O_7_ and K_2_CrO_4_ (500 µgml^−1^)) on the growth of plants inoculated with bacterial isolates was observed by comparing their growth parameters with the non-treated plants. Certified seeds of *Lens culinaris* (var. masoor-2009) were procured from a Government seed organization (Punjab Seed Corporation, Lahore) and these seeds were then surface sterilized to remove dust and other bacteria by treating these seeds with standard solution of 0.1% HgCl_2_ for 3 to 4 min and then washed several times with autoclaved distilled water. These sterilized lentil seeds were treated with chromium-tolerant bacterial liquid culture (inoculum) after adjusting the optical density of the culture to 10^6^–10^7^ CFU ml^−1^ at 600 nm. For control treatment, seeds were treated with autoclaved distilled water for the same period of time. The inoculated seeds of *Lens culinaris* (var. masoor-2009) were spread in a uniform manner in autoclaved petri-plates on sterilized filter paper with the help of forceps. Ten ml of both chromium salt solutions i.e., K_2_Cr_2_O_7_ and K_2_CrO_4_ with varying concentrations (0, 50, 100, 200, 400 and 500 µgml^−1^) was supplied to each respectively labeled autoclaved petri-plate containing sterilized seeds separately. Petri-plates were kept in dark at 25 ± 2 °C for three days. After germination, germinated seedlings were transferred to the labeled pots each containing about 140 gm autoclaved sieved soil i.e., seven seeds per pot and chromium stress solution with varying concentration (0, 50, 100, 200, 400 and 500 µgml^−1^) of both chromium salts (K_2_Cr_2_O_7_ and K_2_CrO_4_ ml^−1^) were given to the respective labeled pots separately. The pots were placed in light (10 K lux, 16 h duration) at 25 ±  2 °C. After 21 days of growth, plants were harvested and different growth parameters recorded which include shoot length, root length, fresh weight and number of leaves of plants, auxin content, pigment content and protein content. The experiment was repeated thrice. Auxin content, pigment content and protein content were estimated by following Mahadevan (1984), Lichtenthaler & Wellburn (1983) and Lowry, Resebrough & Farr (1995), respectively.

## Results

### Screening of chromium-tolerant auxin-producing isolates and their characterization

**Figure 1:**
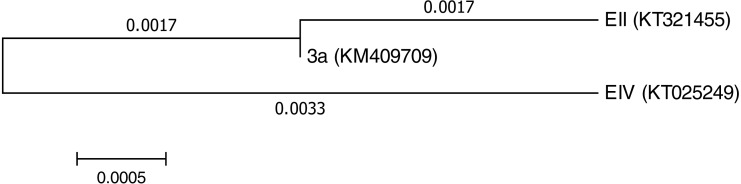
Phylogenetictree showing evolutionary relationship of the isolates (*Bacillus cereus* (3a), *Bacillus anthracis* (EII) and *Bacillus cereus* (EIV)).

**Figure 2:**
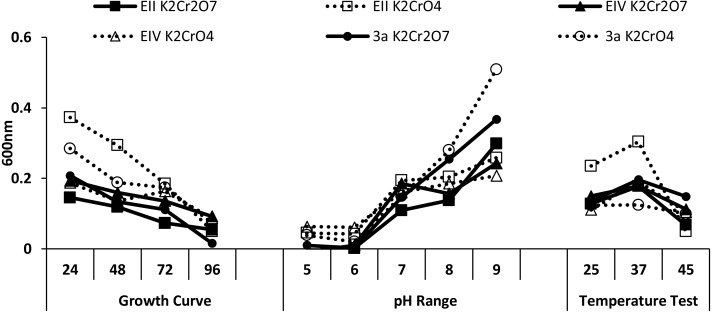
Growth evaluation of isolates after different incubation time (24–96 h), pH (5–9) and temperatures (25, 37 and 45 ° C) under stress condition.

Three bacterial isolates (EII, EIV and 3a), on the basis of their ability to produce auxin and chromium-resistance potential, were selected for the present study. 16S rDNA sequencing of the bacterial species named as EII, EIV and 3a was carried out and the sequences obtained were then submitted to GenBank. The bacterial isolate EII has shown 98% similarity to *Bacillus anthracis* with accession number KT321455 while the other two isolates (EIV and 3a) have shown 99 and 96% homology with *Bacillus cereus*. These bacterial isolates were given accession numbers i.e.,  KT025249 and KM409709, respectively (Fig. 1). All the three selected bacterial cultures have shown highest growth rate at 37 °C after 24 h of incubation period in the presence of K_2_Cr_2_O_7_ and K_2_CrO_4_ i.e., 500 µgml^−1^. Growth of all the isolates was better in medium with neutral pH but maximum growth was noted with medium having alkaline pH (Fig. 2).

### Interactions of bacterial isolates with *Lens culinaris*

**Figure 3:**
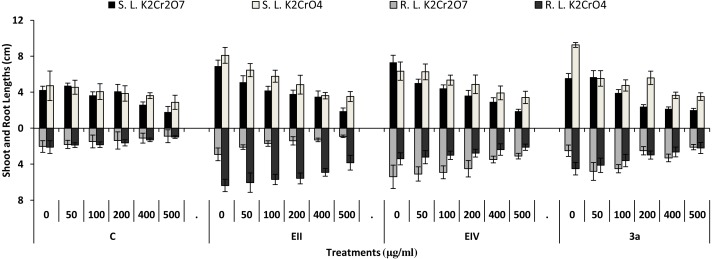
Effect of bacterial inoculations (K_2_Cr_2_O_7_ and K_2_CrO_4_(0–500 µgml^−1^)) on SL and RL (cm) of *Lens culinaris* (C, without bacterial inoculation; EII, EIV and 3a, bacterial isolates).

**Figure 4:**
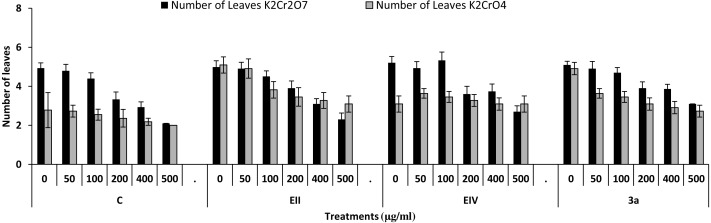
Effect of bacterial inoculations (K_2_Cr_2_O_7_ and K_2_CrO_4_(0–500 µgml^−1^)) on number of leaves of *Lens culinaris* (C, without bacterial inoculation; EII, EIV and 3a, bacterial isolates).

**Figure 5:**
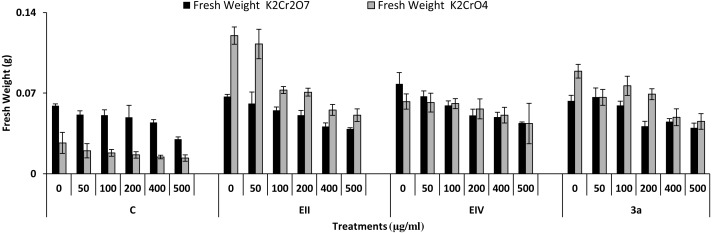
Effect of bacterial inoculations (K_2_Cr_2_O_7_ and K_2_CrO_4_ (0–500 µgml^−1^)) on fresh weight (g) of *Lens culinaris* (C, without bacterial inoculation; EII, EIV and 3a, bacterial isolates).

**Figure 6:**
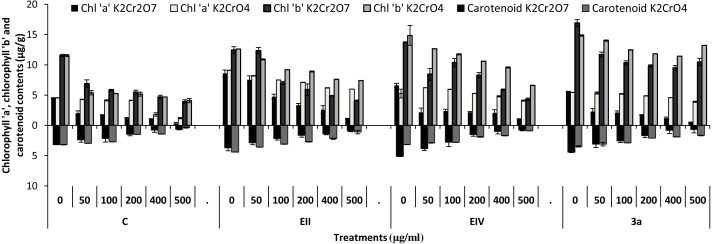
Bacterial inoculations effect (K_2_Cr_2_O_7_ and K_2_CrO_4_ (0–500 µgml^−1^)) on chlorophyll ‘a’, ‘b’ and carotenoid content (µgg^−1^) of *Lens culinaris* (C, without inoculation; EII, EIV and 3a, bacteria).

**Figure 7:**
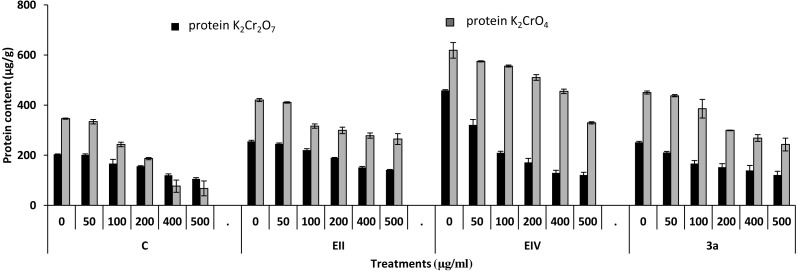
Effect of bacterial inoculations (K_2_Cr_2_O_7_ and K_2_CrO_4_ (0–500 µgml^−1^)) on protein content (µgg^−1^) of *Lens culinaris* (C, without bacterial inoculation; EII, EIV and 3a, bacterial isolates).

**Figure 8:**
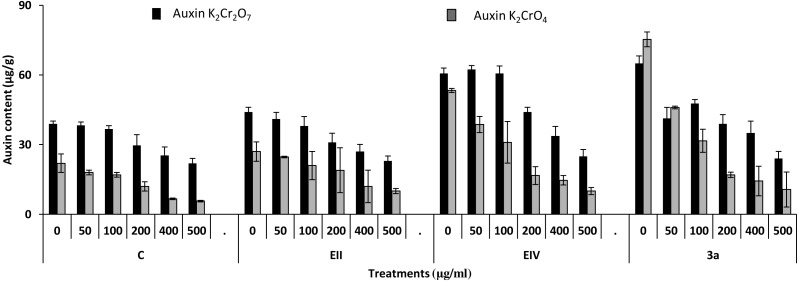
Effect of bacterial inoculations (K_2_Cr_2_O_7_ and K_2_CrO_4_ (0–500 µgml^−1^)) on auxin content (µgg^−1^) of *Lens culinaris* (C, without bacterial inoculation; EII, EIV and 3a, bacterial isolates).

In the plant growth experiment, presence of chromium stress induced prominent reduction by lowering the total fresh weight of the treated plants, their number of leaves and overall plant height (shoot length) in both inoculated and non-inoculated treatments. In the absence of chromium, all the inoculated plants have shown 70 and 80% enhancement in length of plant shoot with selected isolates i.e., *B. cereus* (EIV) and *B. cereus* (3a), respectively, over control treatment. Increase in concentration of chromium from 0–500 µgml ^−1^ of K_2_Cr_2_O_7_ and K_2_CrO_4_ also resulted in significant reduction of plant shoot length. Severe damage was observed in case of chromium stress of K_2_Cr_2_O_7_ but bacterial inoculation with *Bacillus anthracis* (EII) caused significant increase in shoot length of the treated plants from 50 –500 µgml^−1^ i.e., 24, 17, 12, 35 and 6% in case of K_2_Cr_2_O_7_ as compared to treatments with K_2_CrO_4_ affected plants when compared with respective controls. The effect of chromium toxicity was considerably higher in plant roots than in plant shoots for both bacterial inoculated and non-inoculated treatments exposed to chromium in higher amount (Fig. 3). Prominent reduction in root length was noted due to toxic effects of chromium stress from 50–500 µgml^−1^ in non-inoculated treatments. However, the root length of lentil plants significantly increased by 150 and 115% as a result of bacterial treatment with isolates *B. cereus* (EIV) and *Bacillus cereus* (3a) respectively, when results were compared with non-inoculated control plants. Similarly under chromium stress, treatment with *B. cereus* (EIV) caused 177, 233, 231, 216 and 248% increment in root length with stress of K_2_Cr_2_O_7_ (50, 100, 200, 400, 500 µgm l^−1^) respectively. Similarly, *B. anthracis* (EII) caused 221, 211, 250, 427 and 352% proliferation in root length of treated plants under K_2_CrO_4_ stress from 50, 100, 200, 400 and 500 µgm l^−1^ respectively, over non- inoculated control. Similar enhancements were noticed in the number of leaves i.e., 85, 6 and 81%, respectively, when lentil plants were grown in the absence of chromium (Fig. 4). On the other hand, when *Bacillus anthracis* (EII) was used for inoculation under chromium stress, the number of leaves of inoculated lentils were significantly increased by 11, 21, 9, 28 and 29% (with K_2_Cr_2_O_7_) and 81, 52, 44, 51 and 55% (with K_2_CrO_4_) ranging from 50, 100, 200, 400 and 500 µgm l^−1^ respectively, when compared with plants without inoculation treatment under the identical experimental conditions. This indicates chronic damage of infrastructure of lentil plants due to K_2_Cr_2_O_7_ contamination as compared to K_2_CrO_4_ as number of leaves were increased in case of plants with Potassium chromate stress. Similar findings were observed in fresh weight of *Lens culinaris* in inoculated and non-inoculated treatments in the absence and presence of K_2_Cr_2_O_7_ and K_2_CrO_4_ stress (Fig. 5). Fresh weight of the treated plants also showed significant increase i.e., 300% when treated with *B. anthracis* (EII) as compared to non-inoculated control without stress. While, when lentil plants were inoculated with *B. cereus* (EIV) and *B. anthracis* (EII) in the presence of chromium, the fresh weights of the inoculated lentil plants significantly increased by 31, 16, 0, 7 and 47% (K_2_Cr_2_O_7_) and 450, 289, 213, 257 and 285% (K_2_CrO_4_), respectively, under uniform experimental conditions (Fig. 5). Significant enhancements in fresh weight of lentil plants grown under chromium stress of K_2_CrO_4_ indicate less toxic nature of chromium salt as compared to K_2_Cr_2_O_7_. Similar results were obtained in the number of leaves with treatment of all the three bacterial isolates EII, EIV and 3a, by 125, 65 and 40%, respectively, when plants were grown in the absence of chromium (Fig. 4). On the other hand, when treatment with *B. anthracis* (EII) was applied with chromium, the amount of chlorophyll ‘a’ in treated plants significantly increased by 275, 179, 212, 158 and 233% (with K_2_Cr_2_O_7_) and 91, 82, 73, 246 and 399% (with K_2_CrO_4_) in stress concentrations of 50, 100, 200, 400 and 500 µgml^−1^ respectively, as compared with plants without inoculation treatment under the same conditions (Fig. 6). Chlorophyll b’ content was also improved by 46% as a result of bacterial treatment with *Bacillus cereus* (3a) compared to control in the absence of chromium. Chlorophyll b’ content of the treated plants also showed significant increase in the presence of both chromium salts (i.e., K_2_Cr_2_O_7_ and K_2_CrO_4_), as a result of bacterial treatment with *B. cereus* (EIV) under chromium stress of K_2_Cr_2_O_7_ in concentrations ranging from 50,100, 200, 400 and 500 µgml^−1^ i.e., 135, 125, 108, 109 and 61% respectively, as compared to control. On the other hand, inoculation treatment with *B. cereus* (3a) in the presence of chromium resulted in significant increase in the chlorophyll ‘b’ content of the inoculated lentil plants by 70, 78, 78, 102 and 87% (K_2_Cr_2_O_7_) and 158, 140, 131, 149 and 90% (K_2_CrO_4_) respectively, when compared with non-inoculated plants (Fig. 6). The amount of carotenoid content was considerably greater for inoculated plants grown without chromium (Fig. 6). The carotenoid content of inoculated lentil plants significantly increased by 65% as a result of bacterial treatment with *B. cereus* (EIV) when compared with non-inoculated plants. However, under chromium stress, treatments with *Bacillus cereus* (3a) and *Bacillus cereus* (EIV) caused almost similar enhancements i.e., 62, 28, 1, 24 and 23% increase (under chromium stress of K_2_Cr_2_O_7_) and 24, 15, 82, 55 and 206% increase (under chromium stress of K_2_CrO_4_) in carotenoid content with chromium stress ranging from 50.100, 200, 400 and 500 µgml^−1^ respectively. In the absence of chromium stress, treatment with bacterial isolate *Bacillus cereus* (EIV) caused significant improvement of protein content (126%) with respect to control. Plants grown in the presence of hexavalent chromium caused significant stress on lentil seedlings (Fig. 7). Bacterial inoculation with *B. cereus* (EIV) caused increase in protein content upto 60, 26, 10, 9 and 15% with addition of K_2_Cr_2_O_7_ and 72, 129, 174, 498 and 383% with addition of K_2_CrO_4_ in concentration ranging from 50, 100, 200, 400 and 500 µgml^−1^ respectively, when compared with control. Microbial inoculation caused significant augmentation in the total amount of auxin in bacterial-treated plants when they were compared with their respective control treatments. Increase in chromium concentration of both K_2_Cr_2_O_7_ and K_2_CrO_4_ from 0 to 500 µgml^−1^ caused reduction in auxin content as compared to respective control treatment. Seedling length of the treated plants was again increased by bacterial inoculation. The amount of auxin produced by inoculated plants significantly increased upto 241% as a result of bacterial treatment with *B. cereus* (EIV) over respective control under similar conditions. However, under chromium stress, treatment with *B. cereus* (EIV) caused 63, 69, 52, 36 and 14% increment in auxin content with chromium stress of K_2_Cr_2_O_7_ (50–500 µgml^−1^) respectively. Moreover, under chromium stress of K_2_CrO_4_, inoculation with *Bacillus cereus* (EIV) caused 117, 82, 42, 114 and 67% increase and *B. cereus* (3a) exhibited 156, 88, 42, 100 and 83% enhancement in auxin content of treated plants under stress condition ranging from 50, 100, 200, 400 and 500 µgml^−1^, respectively, over non-inoculated control (Fig. 8).

## Discussion

In the present research, three bacterial isolates i.e., EII, EIV and 3a were selected after checking their auxin producing potential and ability to tolerate chromium. These isolates were identified as *B. anthracis* (EII), *B. cereus* (3a) and *B. cereus* (EIV) through 16S rDNA sequencing (Fig. 1). All these strains were characterized physiologically in the presence of two different chromium salts i.e., K_2_Cr_2_O_7_ and K_2_CrO_4_ (500 µgml^−1^). Temperature and pH both play an important role in the regulation of variation in environmental conditions that may cause physiological changes in plants. All the three liquid cultures of selected strains have shown their highest growth in growth medium at 37 °C with pH 9 after 24 hours of incubation in the presence of both chromium salts i.e., K_2_Cr_2_O_7_ and K_2_CrO_4_(500 µgml^−1^). Maximum bacterial growth at pH 9 showed the alkaline nature of these bacterial species. Similar growth rates in alkaline environments have also been reported by other workers (Anuar & Chan, 2017; Alpaslan et al., 2017; Ahmad et al., 2018). Higher temperature causes reduction in bacterial growth due to breakdown of membrane lipids and disturbance in production of cellular proteins which results in breakdown of bacterial cells (Ahmed, Sabri & Hussnain, 2004; Singh, Verma & Gaur, 2016). Bacterial inoculation has improved the shoot length of inoculated plants in both treatments (K_2_Cr_2_O_7_ and K_2_CrO_4_) in the presence and absence of stress of chromium. Similarly, microbial treatment also caused enhancement in root length of the treated plants which may be due to ability of roots exudates to produce auxin. This auxin production potential is a key factor in root enhancement which resulted in increase in roots lengths of the treated plants (Belimova et al., 2015). Maximum increase in leaf number was also noted with bacterial inoculation but excellent results were obtained by inoculation of *B. cereus* (EIV) i.e., 150% in the absence of chromium (Fig. 4). Ethylene production was inhibited in the roots of treated plants due to the presence of abiotic stress. This is due to the activity of enzyme named as 1-aminocyclopropane-1-carboxylate (ACC) deaminase which utilizes NH_3_ and acts as a source of nitrogen. Activity of ACC deaminase results in decreased ACC production in the plants due to deleterious effects of chromium metal in the plant cells (Ahmed & Hasnain, 2010). Increase in the amount of nitrogen and proliferation of root nodules as a result of treatment with bacteria may be linked to bacterial activity and observed in the form of increased plant vigor which was more prominent in case of K_2_CrO_4_ chromium stress (Gupta et al., 2018). A significant increase in fresh weight was also recorded as a result of treatment with *B. anthracis* (EII) in the absence and presence of chromium stress (Fig. 5). Yasin et al. (2015) also reported enhanced fresh weight as a result of bacterial inoculation under heavy metal stress. In another study, similar results have been reported in mung bean growth observed under *P. vermicola* inoculation in the presence of copper stress (Yasin et al., 2015). Inoculated mung bean plants had caused improvement in total chlorophyll content and in nodule number per plant as compared to inoculated plants with copper stress. These findings suggest that treated plants have developed different defense mechanisms in order to scavenge the peroxidase and free radicals coming from chromium metal. These protective mechanisms include different antioxidant enzymes and some non-enzymatic antioxidative complexes. The available literature describes both decreasing and increasing trends in their activity, depending on the species of the plant, its plant organ, type of metal and its concentration, duration of the stress, age of the plant and growth medium. The results of photosynthetic pigments including chlorophyll a, chlorophyll b and carotenoid contents of the treated plants showed that among all the chromium-tolerant rhizobacterial isolates, *B. anthracis* (EII) showed highest increment in the chlorophyll a, b and carotenoid contents, in different chromium stresses i.e., 50–500 µgml^−1^ of K_2_Cr_2_O_7_ and K_2_CrO_4_, compared to non-inoculated control which may be due to ACC-deaminase and nitrogen fixing ability (Fig. 6). The overall trend regarding growth potential of other rhizobacterial isolates under chromium stress of both salts was in the order 3a>EIV. However, toxicity of K_2_Cr_2_O_7_ was more pronounced and severe damage was observed in all the growth and biochemical parameters of lentil plants grown under stress of K_2_Cr_2_O_7_ as compared to K_2_CrO_4_. Hassan et al. (2016) also observed PGPR inoculation effect on the growth of a bean plant and found that inoculation treatment with PGPR caused considerable increase in the photosynthetic pigment of the runner bean. In the present study, all the three bacterial strains proved to be auxin producers, but *Bacillus cereus* (3a) resulted in significant production of auxin (67 and 241%) in chromium-free as well as chromium supplemented growth media. Bacterial isolate *B. cereus* (EIV) also showed a higher auxin production (114% at 400 µgml^−1^) in chromium supplemented media rather than media without chromium (Fig. 8). Ahmed, Sabri & Hussnain (2004) also reported root exudates as a source of tryptophan precursor for auxin production for plant-associated bacteria. This auxin production potential conferred by all studied strains showed their plant growth-enhancing ability in chromium polluted environment. In the present study, the comparison among the deleterious effects of both chromium salts i.e., K_2_Cr_2_O_7_ and K_2_CrO_4_ have been illustrated in order to understand the potential threats associated with the use of these two chromium salts by different industries. The proposed experiment indicates that plants were able to grow in the presence of both chromium salts, K_2_Cr_2_O_7_ and K_2_CrO_4_ i.e., 500 µgml^−1^. However, plant growth of lentil seedlings was more pronounced in case of K_2_CrO_4_ as compared to K_2_Cr_2_O_7_, which indicates lethal toxic nature of K_2_Cr_2_O_7_.

## Conclusions

Microbial assisted treatment of agricultural crops with PGPR is a promising approach to utilize chromium contaminated soils and to increase the area of available land for agricultural practices. PGPRs protect the plants from deleterious effects of heavy metals like chromium by producing certain phytohormones and several antioxidant enzymes thereby converting Cr (VI) into Cr (III) which is less toxic, less mobile and insoluble. Bacterial isolate *Bacillus cereus* (EIV) exhibited best growth production potential under toxic chromium stress upto 500 µgml^−1^ in both chromium treatments of K_2_Cr_2_O_7_ and K_2_CrO_4_. It is very important to increase the productive efficiency and working potential of a specific PGPR to reduce toxic effects of different heavy metals by establishing resistance mechanism with the proper acclimatization and optimization stability. This study illustrates greater toxicity with enhanced deleterious impact of K_2_Cr_2_O_7_ as compared to K_2_CrO_4_ on plants, therefore, use of chromium in the form of K_2_Cr_2_O_7_ in the industrial processes should be replaced with K_2_CrO_4_ which is less toxic and less hazardous as compared to K_2_Cr_2_O_7_.

##  Supplemental Information

10.7717/peerj.6076/supp-1 Supplemental Information 1 Supplemental Data

## Additional Information and Declarations

### DNA Deposition

The following information was supplied regarding the deposition of DNA sequences: 16S rDNA sequencing of the isolates EII, EIV and 3a was carried out and the sequences obtained were then submitted to GenBank. The isolate EII has shown 98% similarity to isolate Bacillus anthracis with accession number KT321455 while the two isolates EIV and 3a have shown 99 and 96% homology with Bacillus cereus and were given accession numbers KT025249 and KM409709 respectively.

### Data Availability

The following information was supplied regarding data availability: Raw data is provided in Supplemental Files.
